# Regulatory T cell therapy in autoimmune liver disease and transplantation

**DOI:** 10.1016/j.jhepr.2025.101394

**Published:** 2025-03-12

**Authors:** Maegen Fleming, Alberto Sanchez-Fueyo, Niloufar Safinia

**Affiliations:** Roger Williams Institute of Liver Studies, Department of Inflammation Biology, School of Immunology and Microbial Sciences, James Black Centre, King's College London, London, SE5 9NU, United Kingdom

**Keywords:** Autoimmune liver disease (AILD), Liver transplantation, Regulatory T cells (Tregs), Adoptive cell therapy, Chimeric antigen receptor (CAR), In vivo modulation, Low-dose IL-2, Antigen specific Tregs, Clinical Trials

## Abstract

In this review, we explore the application of regulatory T cells (Tregs) as cellular therapies in the setting of autoimmune liver diseases and liver transplantation. At present, there are limited treatment options for end-stage liver disease, which in many cases requires transplantation. Following liver transplantation, immunosuppressive drugs are administered, often throughout the course of the patient’s life, to prevent organ rejection. When used for a prolonged period, immunosuppressive drugs can have detrimental effects on the patient’s health and quality of life. Tregs are an attractive target for cellular therapy in this context because of their innate ability to control inflammatory immune responses. In this review, we cover the different generations of Treg-based therapies, their potential roles in the treatment of autoimmune liver diseases and transplantation, and the future outlook for Tregs as cellular therapies.

## Graphical abstract

**Figure:**
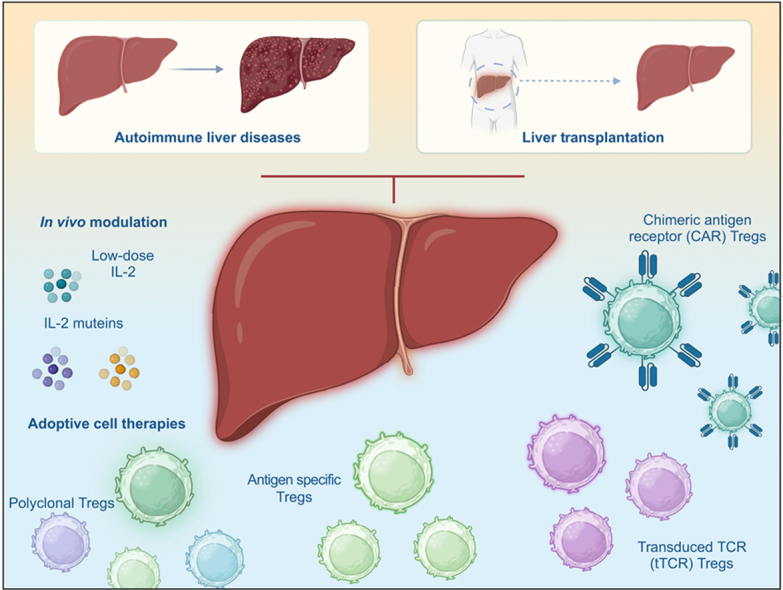

**Keypoints**

• The global burden of liver disease continues to rise, yet therapeutic options remain limited and current treatments often entail significant adverse effects.
• Tregs function as potent suppressors of inflammation, which is a principal mediator of hepatic injury.
• While the pathogenesis of autoimmune liver diseases – encompassing AIH, PBC, and PSC – remain incompletely understood, they share characteristics with other autoimmune conditions, notably the presence of self-reactive effector immune cells that represent potential targets for Treg-based interventions.
• In liver transplantation, allograft rejection is partially mediated by recipient immune cell reactivity, which can be attenuated through the immunomodulatory actions of tolerance-inducing Tregs.
• Therapeutic modulation of Tregs can be achieved through multiple approaches, including *in vivo* expansion via low-dose IL-2 administration or the delivery of *ex vivo-*expanded Treg populations.
• Clinical trials evaluating Treg-based therapeutic strategies, including low-dose IL-2 administration and adoptive Treg transfer, have established favourable safety profiles in patient populations.
• Engineered Tregs offer the potential for enhanced target specificity, stability, and therapeutic potency compared to their unmodified counterparts, positioning technologies such as CAR Tregs at the forefront of Treg-based therapeutic development.

## Introduction

The escalating global burden of liver disease, reflected in increasing prevalence and mortality rates, has intensified the need for innovative therapeutic interventions. While conventional immunosuppressive agents, including corticosteroids, anti-metabolites, and calcineurin inhibitors, have transformed solid organ transplantation and demonstrated efficacy in autoimmune conditions, their long-term administration is associated with significant adverse effects. This therapeutic limitation has catalysed research into alternative strategies capable of achieving sustained immunological tolerance. Cell-based therapeutic approaches have emerged as promising candidates for inducing and maintaining immune homeostasis without the need for chronic immunosuppression. Among potential cellular therapeutics, including tolerogenic dendritic cells and regulatory macrophages, regulatory T cells (Tregs) have emerged as the most extensively characterised immunoregulatory cell population.

In this comprehensive review, we evaluate the current landscape of Treg-based cellular therapies, encompassing technological innovations, clinical trial outcomes, and emerging developments. Particular emphasis is placed on next-generation engineered Treg products, incorporating chimeric antigen receptor (CAR) technology, that have been designed to enhance organ specificity and therapeutic efficacy.

## Regulatory T cells

While multiple T-cell subsets exhibit immunoregulatory functions, including suppressive Tr1 cells, T helper (Th)3 cells, γ/δ TCR-expressing T cells, CD8^+^CD28^−^ T cells, and regulatory Th2 cells^1^ – the designation "regulatory T cell" (Treg) predominantly refers to T cells characterised by the expression of CD4, CD25, and the transcription factor Forkhead-box protein 3 (FOXP3).

CD4^+^CD25^+^FOXP3^+^ Tregs comprise approximately 1-10% of the circulating CD4^+^ T cell population and are instrumental in resolving inflammatory responses and maintaining peripheral tolerance. These cells are distinguished by constitutive high expression of the interleukin-2 receptor α chain (CD25) and the lineage-defining transcription factor FOXP3, which is essential for Treg development and function. The critical importance of FOXP3 is demonstrated by mutations in the *FOXP3* gene, which result in severe Treg dysfunction and manifest as immune dysregulation, polyendocrinopathy, and enteropathy, X-linked syndrome (IPEX),^2^^,^^3^ a severe multiorgan autoimmune condition. Moreover, single nucleotide polymorphisms in Treg-associated genetic loci contribute to functional abnormalities implicated in polygenic autoimmune disorders, including type 1 diabetes and rheumatoid arthritis.^4^

**Fig. 1:**
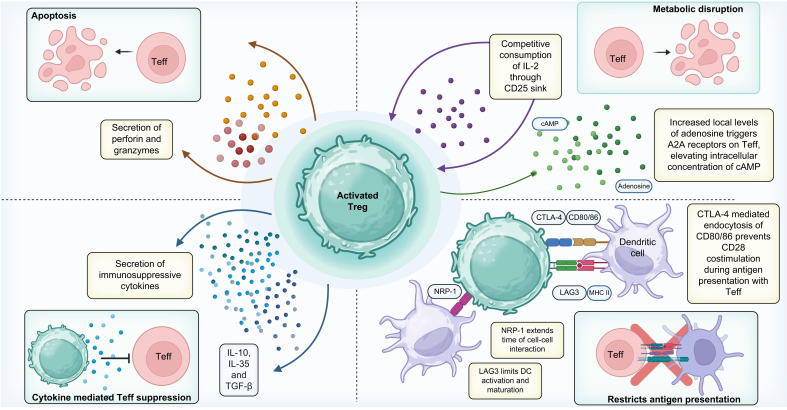
Mechanisms of Treg suppression. Tregs mediate immunosuppression through a multitude of pathways. These include inducing apoptosis in Teffs through the secretion of perforin and granzymes A and B or through metabolic disruption through IL-2 inhibition. Tregs also possess a higher intracellular level of cAMP which is detrimental to Teff function. Immunosuppressive cytokines such as IL-10, IL-35 and TGF-β, secreted by Tregs, impede Teff function and promote the conversion of dendritic cells and naïve CD4^+^ to tolerogenic cells. DCs play a key role in Teff activation, therefore they are a key target of Tregs. Tregs also secrete cytotoxic granules to induce apoptosis in autologous target cells such as CD4^+^ and CD8^+^ effector T cells.^7^ Tregs competitively consume IL-2 through CD25, depleting local levels for Teffs. Inhibition of IL-2 disrupts Teff metabolism leading to Bim-mediated apoptosis. Additionally, Tregs release adenosine which is taken up by Teffs through the A2A receptor and is used to produce cAMP, which has a suppressive effect on Teffs.^8^ Metabolic disruption is also mediated through the ectonucleotidase CD39. CD39 activates a hydrolysis cascade that facilitates the formation of immunosuppressive adenosine from pro-inflammatory ATP.^9^ Tregs use surface protein CTLA-4 to bind and endocytose CD80/86 on the surface of antigen-presenting cells. This binding leads to T-cell suppression, as CD80/86 is required for CD28 costimulation and thus T-cell activation.^10^ Antigen presentation is also reduced by Tregs through surface molecules NRP-1 and LAG3, which are used to prevent dendritic cell maturation. Teffs, effector T cells; Tregs, regulatory T cells. All figures Created in BioRender. Fleming, M. (2025) https://BioRender.com/q45x981.

Tregs migrate to inflammatory sites, which is governed by T-cell receptor (TCR) specificity and chemokine receptor expression patterns. Their immunosuppressive function is initiated through TCR-mediated activation and costimulatory signalling, notably via CD28. Tregs employ diverse mechanisms to suppress multiple immune cell populations, including pro-inflammatory effector T cells (Teffs), dendritic cells, natural killer (NK) cells, and B cells, through both contact-dependent and soluble mediator-dependent pathways (Fig. 1).

The immunoregulatory capacity of Tregs extends beyond their direct antigen-specific interactions, exhibiting broader suppressive effects through bystander suppression, a phenomenon whereby Tregs modulate the function of proximal immune cells regardless of their antigen specificity or direct cellular contact.^5^^,^^6^

### Treg development

Tregs are classified according to their site of development: thymic/natural or peripheral/induced. As their name suggests, FOXP3^+^ thymic Tregs (tTregs) form within the thymus, maturing from double positive CD4^+^ and CD8^+^ T cells that bind strongly to self-antigens.^11^ They play a key role in immune tolerance and homeostasis. Peripheral Tregs (pTregs) develop from naïve CD4^+^ conventional T cells in the periphery, where FOXP3 expression is induced through stimulatory signals including IL-2 and IL-10. pTregs predominantly act at sites of inflammation, particularly at the mucosae, and have been best characterised within the gut. There are still some discrepancies surrounding the conditions and factors that influence the generation of pTregs. Studies have shown that non-immunogenic methods of antigen administration or sub-immunogenic antigen doses were optimal for pTreg induction, suggesting that antigen-presenting cells play a key role in this conversion of conventional T cells.^12^^,^^13^

Further studies into FOXP3^+^ CD4^+^ T cells have elucidated subgroups of FOXP3-expressing cells with different phenotypic and functional properties, not all of which are suppressive. FOXP3^+^ Tregs can be subdivided into resting CD45RA^+^ FOXP3lo, activated CD45RA^-^ FOXP3hi and non-suppressive CD45RA^-^ FOXP3lo T cells.^14^ CD45RA^+^ Tregs cells are regarded as naïve T cells. Although they form the largest proportion of Tregs in cord blood, they decline steadily with age, with CD45RA^+^ Tregs making up less than 10% of FOXP3^+^ Tregs in young adults. In humans, the majority of Tregs are CD45RO^+^ which is a marker of previous activation and proliferation.^15^ CD45RO^+^expressing Tregs are predominantly found in adult peripheral blood and have been shown to exhibit limited replicative ability, whilst being more prone to apoptosis.^16^ When expanded *in vitro*, a proportion of CD45RO^+^ Tregs also display a loss of FOXP3 expression and suppressive function. CD45RA^+^ Tregs, on the other hand, can produce a rapidly expanding homogenous population *in vitro.*

### Treg plasticity

Stable FOXP3 expression is governed by several factors including epigenetic modifications such as histone acetylation and CpG demethylation. The demethylation of a particular area within the *FOXP3* locus known as the Treg-specific demethylated region has been shown to be involved in the regulation of FOXP3 expression and maintenance of Treg lineage *in vitro*.^17^

**Fig. 2:**
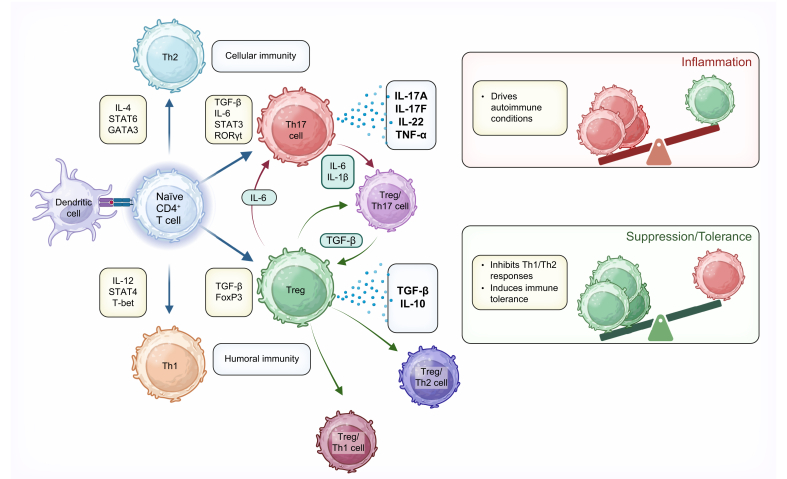
The dynamic balance between Tregs and Th cells. Naïve CD4^+^T cells differentiate into different T-cell lineages based on cytokine and transcription factor signalling at the time of activation. Tregs do not always remain committed to their pathway and under different conditions can convert to cells that possess characteristics of other Th cells. Th, T helper; Tregs, regulatory T cells. All figures Created in BioRender. Fleming, M. (2025) https://BioRender.com/q45x981.

Without robust FOXP3 expression to reinforce Treg lineage commitment, Tregs convert or partially convert to an inflammatory phenotype (Fig. 2). Treg conversion and stability is believed to be largely controlled by environmental stimuli such as cytokines. When Tregs are exposed to an inflammatory environment they have been shown to exhibit lineage instability and adopt T helper cell characteristics.^18^ In murine- and human-derived tTregs, treatment with Th17-promoting cytokines IL-6, IL-1β and IL-23 resulted in production of IL-17 and the destabilization of FOXP3.^19^ A skew towards Teffs, particularly Th17 cells, is associated with inflammation and the onset and/or progression of autoimmunity. Therefore, Treg phenotypic shifting may contribute to the maintenance of pro-inflammatory immune cells, highlighting the importance of Treg phenotype stability.

### Tissue-resident Tregs

Tregs demonstrate distinct patterns of tissue distribution, encompassing both circulating populations in peripheral blood and resident populations within non-lymphoid organs. These tissue-resident Tregs undergo progressive adaptation in response to local inflammatory and metabolic cues within their respective microenvironments.^20^ Comprehensive tissue distribution analysis by Burton *et al.*, examining 48 distinct murine tissues, demonstrated the ubiquitous presence of Tregs across tissues, with non-lymphoid non-gut tissues^21^ collectively harbouring 0.3% of the total Treg population. While the precise molecular mechanisms governing tissue-specific Treg adaptation remain to be fully elucidated, substantial evidence indicates that tissue-resident Tregs play crucial roles in maintaining local homeostasis and facilitating tissue repair processes.

In humans, Tregs have been shown to reside in adipose tissue (particularly visceral adipose tissue), skin, colon, brain, uterine tissue, muscle and liver.[110], [111], [112], [113], [114], [115] Murine intrahepatic Tregs have been shown to persist in lower numbers than in other sites such as blood, adipose tissue, lung, secondary lymphoid organs and the thymus.^22^ Additionally, intrahepatic Tregs display a distinct transcriptional and phenotypic profile. These include increased expression of tissue regenerative transcripts such as *Ace* and *Fn1*. Although the extent of their role in the liver is unclear, these profiles are suggestive of tissue-specific adaptations.

## Insights into the role of Tregs in autoimmune liver disease

Recent studies have highlighted the involvement of Tregs in liver pathologies. An increase in intrahepatic Treg numbers was observed during chronic viral hepatitis and hepatocellular carcinoma.^23^ This influx of Tregs is associated with the suppression of effector immune cells responsible for clearing viral infection as well as cancer surveillance/killing. In contrast, it has been well established that Treg depletion and dysfunction are linked to the onset of autoimmune disease in different tissues.^24^

Ye Oo *et al.* established that Tregs actively migrate across inflamed hepatic sinusoidal endothelium through a specific mechanism: recognition of IFN-induced chemokines CXCL9 and CXCL10 via CXCR3.^25^ Their research revealed a robust co-localisation of Tregs with intrahepatic dendritic cells during liver inflammation. The recruitment of Tregs to inflammatory sites is further mediated by dendritic cell-secreted chemokines CCL17 and CCL22, which engage with CCR4 on Tregs. Significantly, the study demonstrated markedly elevated Treg frequencies in chronic inflammatory and autoimmune liver disease tissues compared to healthy liver tissue. However, despite non-compromised migration capabilities, these Tregs failed to suppress effector-mediated immune responses, indicating fundamental dysfunction rather than recruitment defects. The following section examines the distinctive features of autoimmune liver diseases and the critical role of Treg dysfunction in disease progression.

## Autoimmune liver disease

Autoimmune liver diseases are generally divided into three main categories, autoimmune hepatitis (AIH), primary biliary cholangitis (PBC) and primary sclerosing cholangitis (PSC).^26^ Patients can also present overlapping diagnoses for the three diseases. Although the precise aetiology of these ailments remains elusive, the three involve a loss of peripheral self-tolerance to liver-associated antigens and share a common pattern of chronic hepatic injury.^27^

### Characteristics of AIH

AIH is an immune-driven disease, whose onset is most likely triggered by a combination of genetic and environmental factors. A recent meta-analysis by Hahn *et al.* showed that the incidence of AIH is highest in female adults in North America and Oceania, with the general rate of AIH increasing steadily from 1970 to 2019.^28^

AIH can be subdivided into types 1 and 2 according to the serological profile.^29^ AIH is generally characterised by increased serum transaminase and immunoglobulin G levels and the presence of positive circulating autoantibodies.^30^ Patients with AIH type 1 display positive anti-nuclear antibodies and/or anti-smooth muscle antibodies in contrast to those with type 2 AIH, which is associated with positive anti-liver-kidney microsomal type 1 and/or anti-liver cytosol type 1 antibodies.^31^ AIH type 2 also predominantly affects children and adolescents rather than adults.

The autoimmune reaction in AIH is initiated by presentation of a liver antigen to an undifferentiated T cell and the subsequent successful activation of this T cell. In response to activation, naïve T cells will differentiate into effector Th1, Th2 and Th17 cells, which have been implicated in liver damage. The exact impact Tregs exert during AIH is unclear. Data collected from mouse models of AIH display conflicting results regarding the number of Tregs present and their functionality, although it is important to note that differences in AIH induction and Treg detection markers may have contributed.^32^ Similarly, in patients with AIH, the role of Tregs in disease pathology has been difficult to discern. However, the Treg/Th17 ratio has been used to predict the extent of liver inflammation and restoring this intrahepatic balance has been highlighted as a potential therapeutic target. Moreover, studies conducted in a mouse model of AIH, suggested thatlow-dose IL-2 treatments helped rescue intrahepatic homeostasis by restoring the balance of Tregs/Teffs.^33^

### Characteristics of PBC

PBC is a chronic cholestatic liver disease, through which the intrahepatic bile ducts are progressively destroyed. The direct cause of PBC is unknown but its pathogenesis is believed to be multifactorial. Song *et al.* observed that the ratio of Th17 to Treg cells is skewed in favour of pro-inflammatory Th17 cells and that Tregs levels are lower in patients with PBC, compared to healthy controls and patients with chronic hepatitis B.^34^ Mouse models of PBC also exhibit this elevation in Th17 cells. Studies into the effects of low-dose IL-2 on Th17/Treg cell frequencies in mouse models of PBC displayed a decreased Th17 population and increased Treg numbers.^35^ It was also observed that Tregs isolated from patients with PBC are more sensitive to IL-12-mediated differentiation into Th1-like cells, which reduces their suppressive capacity.^36^ This suggests that Treg disruption may play a larger role in PBC progression. Therefore, promoting the expansion of functional Tregs during PBC may be beneficial for slowing or halting disease progression.

### Characteristics of PSC

PSC is a rare disorder, characterised by multi-focal bile duct strictures, caused by inflammation and fibrosis, and progressive liver diseases. As with AIH and PBC, it is most likely brought on by a combination of environmental and genetic factors. Unlike AIH and PBC, PSC predominantly affects males, with the median diagnostic age sitting at 40 years old.^37^ Despite an unclear pathogenesis, it has been thought to include defects in mechanisms protecting against bile acid toxicity, passive leakage of pro-inflammatory microbial components to the portal circulation and potentially an antigenic trigger of microbial origin.^38^ Gut-derived T-cell recruitment to the liver and/or an insult resulting from disturbances in the gut microbiota are also believed to contribute to pathology. Inflammatory bowel disease is commonly present alongside PSC and these patients are at an increased risk of cholangiocarcinoma and colorectal cancer. Currently the only treatment that prolongs life in patients with PSC is liver transplantation.^39^

As seen with PBC, the frequency of Tregs in the peripheral blood of patients with PSC was shown to be reduced when compared to healthy controls.^40^ Interestingly, Treg numbers were also suggested to be lower in these patients with PSC than in patients with PBC. Additionally, the same study observed a decrease in intrahepatic Tregs in patients with PSC at the time of diagnosis. By analysing single nucleotide polymorphisms – identified by genome-wide association studies – in Tregs that correlated with PSC susceptibility, Treg depletion was shown to be associated with genetic polymorphisms in *IL2RA*.^41^^,^^42^

## Tregs in liver transplantation

Graft rejection is initiated by alloreactive T cells, most of which target the donor liver through recognition of the donor’s ‘foreign’ MHC (major histocompatibility complex) antigens.^43^ Alongside their role in maintaining immune tolerance to self, Tregs have also been observed to be involved in transplantation tolerance. This is particularly relevant to liver allografts, which are spontaneously accepted in most rodent models in the absence of immunosuppression.[44], [45], [46] Although this process involves the selective deletion of alloreactive T cells recognising alloantigens expressed in the transplanted liver,^47^ it requires the participation of recipient Tregs as well.^48^ Furthermore, in a rat liver transplantation model in which tolerance does not spontaneously occur, the administration of Tregs with donor specificity has the capacity to induce permanent engraftment of the transplanted liver.^49^ Unlike animal models, liver allografts are not spontaneously accepted in humans. However, liver transplantation is the only human transplantation setting in which a sizeable proportion of patients eventually tolerate their transplanted organ and can completely discontinue immunosuppression (a phenomenon referred to as ‘operational tolerance').[50], [51], [52] Although the mechanisms of operational tolerance in humans remain to be fully elucidated, some observations indicate that, as described in rodents, this is also an active process that requires the involvement of Tregs.^53^ In addition, the administration of an autologous cell product enriched in Tregs has been shown to promote permanent engraftment of kidney allografts in non-human primates,^54^ and to induce operational tolerance in human liver transplant recipients.^55^

## Developing Treg-based immunotherapies

**Fig. 3:**
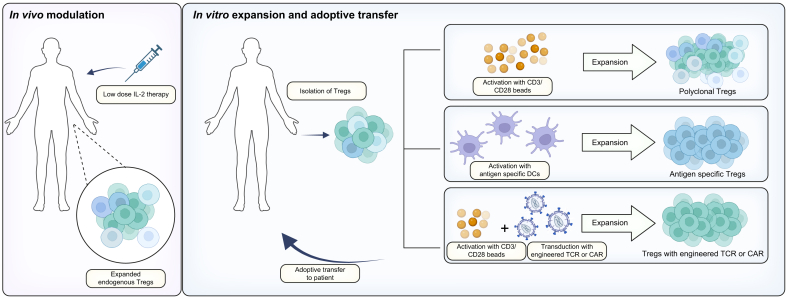
Treg-based therapies. Tregs can be promoted to proliferate *in vivo* through administration of low-dose IL-2. Alternatively, Tregs can be activated through beads or antigen-presenting cells and expanded after being harvested from the patient. The purity and phenotypic stability of the isolated Tregs are vital to producing a successful therapeutic product. Once isolated, Tregs can be activated in different ways to produce various cell products including polyclonal Tregs, antigen-specific Tregs, transduced TCR Tregs and CAR Tregs. CAR, chimeric antigen receptor; DCs, dendritic cells; TCR, T-cell receptor; Tregs, regulatory T cells. All figures Created in BioRender. Fleming, M. (2025) https://BioRender.com/q45x981.

Given their intrinsic suppressive capacity and the evidence supplied by pre-clinical data and early-phase clinical trials, Tregs are an attractive target for cellular therapies. Likewise, the critical role of Tregs in the prevention of autoimmunity and graft rejection supports their curative potential in these settings. Different approaches to Treg-based therapies have been implemented to increase Treg numbers and/or to modify their antigen specificity. Based on the strategies employed to achieve these goals, Treg-based therapies employed in the clinic are commonly classified into three categories. First-generation Treg products involve the expansion of bulk (polyclonal) Treg pools without modifying their antigenic specificity, which can be achieved either *ex vivo* (by isolating circulating Tregs and culturing them in the laboratory with non-specific stimulants), or *in vivo* (by administering Treg growth factors such as low-dose IL-2 or IL-2 muteins directly to patients). Second-generation Treg products are manufactured by culturing Tregs *ex vivo* in the presence of donor alloantigens, so that Tregs with donor reactivity are preferentially expanded. Finally, third-generation Tregs are those that are genetically engineered to acquire donor specificity or additional functional properties. To date, this has mostly involved the use of chimeric antigen receptor (CAR) technology to generate CAR Tregs that recognise a human leukocyte antigen (HLA) molecule exclusively expressed by the transplanted organ (Fig. 3). A summary of the clinical trials employing these different Treg manufacturing strategies is provided in Table 1.

**Table 1:** Liver focused Treg-based therapy clinical trials (https://clinicaltrials.gov search data September 9^th^ 2024).

Therapy	ID	Trial title	Location	Phase	Outcome	References
Expansion of endogenous Tregs in liver transplantation	NCT02949492	Low dose IL-2 *in vivo* modulation of Tregs in liver transplant patients (LITE)	King’s College Hospital, London, UK Sponsor: King’s College London	Phase IV	Terminated (01/2019)	67,94
	NCT02739412	Efficacy of low dose, SubQ interleukin-2 (IL-2) to Expand Endogenous Regulatory T-cells in Liver Transplant Recipients	Beth Israel Deaconess Medical Centre, Boston, Massachusetts, US Sponsor: Beth Israel Medical Centre	Phase II	Completed (07/2022)	95
Expansion of endogenous Tregs in autoimmune hepatitis	NCT01988506	Induction of regulatory T cells by low dose IL-2 in autoimmune and inflammatory diseases (TRANSREG)	Henri Mondor – Méecine Interne Créteil, France Médecine Interne – Hôpital Saint-Antoine Service de médcine vasculaure – HEGP Sponsor: Assistance Publique - Hôpitaux de Paris	Phase II	Completed (04/2021)	96,97
Safety and homing properties of GMP-grade autologous Tregs in AIH	IRAS ID: 177127	Autologous T-regulatory cell tracking after InfUsion in AutoiMmuNe liver disease patients (AUTUMN)	University of Birmingham Sponsor: Medical Research Council	Phase 0	Started (08/2016) Completed (08/2017)	98
Adoptive cell therapy (polyclonal) in liver transplantation	NCT02166177	Safety of adoptive transfer of Tregs in liver transplant patients ThRIL,	King’s College Hospital, London, UK	Phase I/II	Completed (01/2019)	99
Adoptive cell therapy (antigen specific) in liver transplantation	NCT02474199	Safety of donor alloantigen reactive Tregs to facilitate minimization and/or discontinuation of immunosuppression in adult liver transplant recipients (ARTEMIS)	US multicentre trial Sponsor: UCSF	Phase I/II	Started (06/2016) Completed (12/2019)	100,101
Adoptive cell therapy (antigen specific) in liver transplantation	NCT01624077	Safety study of using regulatory T cells induce liver transplantation tolerance	Nanjing Medical University Nanjing, Jiangsu, China Sponsor: Nanjing Medical University	Phase I	Unknown (Started 12/2014)	102
Adoptive cell therapy, donor alloantigen-specific Tregs in liver transplantation	NCT03577431	Liver transplantation with Tregs at MGH and UCSF (LITTMUS)	US multicentre trial Sponsor: NIH	Phase I/II	LITTMUS UCSF – Terminated (03/2023) LITTMUS MGH-Active (Started 03/2019)	103
Adoptive cell therapy, donor alloantigen-specific Tregs in liver transplantation	NCT02188719	Donor-alloantigen-reactive regulatory T cell (darTregs) in liver transplantation (delta)	US multicentre trial Sponsor: NIAID	Phase I	Terminated (06/2019)	109
Adoptive cell therapy (CAR Treg) in liver transplantation	NCT05234190	Safety and clinical activity of QEL-001 in A2-mismatch liver transplant patients (LIBERATE)	European multi-centre trial Sponsor: Quell Therapeutics	Phase I/II	Active	104

GMP, Good Manufacturing Practice.

### *In vivo* modulation of endogenous Tregs using low-dose IL-2

High-dose IL-2 therapy was initially used to treat cancers such as renal cell carcinoma or melanoma, given that, when administered in high doses, IL-2 promotes the expansion and survival of Teffs.^56^ It was later observed that IL-2 signalling is crucial for Treg development and function. Furthermore, due to both the constitutive expression of the IL-2 receptor alpha chain (CD25) and heightened IL-2 signalling (mediated through STAT5), Tregs are capable of responding to very low concentrations of IL-2.^57^ The administration of low-dose IL-2 (at variable doses, but usually <1-2 million IU/day) is indeed capable of increasing the number of circulating Tregs several fold,^58^ and its use has been explored in multiple clinical trials of immune-mediated disease,^59^^,^^60^ with positive results mostly in chronic graft *vs.* host disease and systemic lupus erythematosus.^61^^,^^62^ Furthermore, to address some of the shortcomings of recombinant IL-2 (short half-life, effects on other immune cell subsets such as natural killer [NK] cells and eosinophils), multiple pharmaceutical companies have investigated the use of IL-2 muteins (*i.e*. modified IL-2 molecules with longer half-life and/or higher selectivity for Tregs).[63], [64], [65] Methods used to develop these IL-2 muteins include decreasing CD122 affinity to increase dependence on CD25, thereby promoting Treg specificity. IL-2 mutein candidates have been tested in clinical trials for autoimmune conditions such as systemic lupus erythematosus.^66^ However, the extent to which IL-2 muteins are truly clinically superior to recombinant IL-2 remains unclear and some of these trials were terminated early.

Recently, Lim *et al.* reported the results of a clinical trial in liver transplantation, exploring the capacity of low-dose IL-2 (administered daily) to induce operational tolerance and allow for the complete discontinuation of immunosuppression.^67^ In contrast to reports on autoimmune diseases, while IL-2 markedly increased circulating Tregs, it did not expand those Tregs with donor specificity. Additionally, low-dose IL-2 did not promote the trafficking of circulating Tregs to the liver, and it modified the allograft’s immune microenvironment by inducing molecular pathways typically associated with T cell-mediated rejection. Ultimately, the trial was terminated after the first six enrolled patients developed rejection and none achieved operational tolerance.

### Adoptive transfer of *ex vivo*-expanded Tregs

#### Polyclonal Tregs

Tregs can be isolated from blood and expanded *ex vivo*, in the presence of anti-CD3/anti-CD28 beads and high-dose IL-2, to produce large numbers of polyclonal Tregs that can be adoptively transferred to patients.^68^ *Ex vivo*-expanded polyclonal Tregs are not antigen specific and possess a range of different TCRs capable of recognising multiple targets (similar to the TCR repertoire from the circulating Tregs used to generate them). As a result, the expectation is that very large numbers of Tregs will be needed, given that only a small percentage will be capable of recognising the target autoantigens or alloantigens.

We have previously shown the safety and functionality of autologous polyclonal Tregs in the setting of liver transplantation as part of the *ThRIL* phase I/II clinical trial (NCT02166177). Participants were administered a single infusion of autologous polyclonal Tregs intravenously 3-12 months post liver transplantation. However, the biological and clinical effects of the infused cells could not be investigated within the trial.^57^

#### Antigen-reactive Tregs

The efficacy of polyclonal Tregs is limited by their lack of antigen specificity. Off target tolerance as a side effect of polyclonal Treg therapy may dampen the immune system in a non-specific manner, potentially increasing the patient’s risk of infection and development of cancer (although no such effects have been observed to date, even when billions of polyclonal Tregs were administered to highly immunosuppressed kidney recipients^69^). A solution to preferentially expand Tregs with a predefined specificity is to add these antigens during the manufacturing step, for instance in the form of antigen-presenting cells from the transplant donor. Two different strategies based on this concept were employed in the ARTEMIS and LITTMUS liver transplant trials. Furthermore, the trial reported by Todo *et al.* used a similar approach, but starting with total peripheral blood mononuclear cells rather than purified Tregs.^70^

The use of antigen-reactive Tregs in autoimmunity is more complex than in transplantation due to the very low frequency of autoantigen-specific T cells/Tregs and the difficulties in identifying causative autoantigens.^71^ This limits the applicability of antigen-reactive Tregs in the context of autoimmune liver diseases, though novel epitopes such as the E2 subunit of the pyruvate dehydrogenase complex have recently been implicated in the pathogenesis of PBC.^72^ Emerging targets such as this may be used to develop new engineered Treg therapies.

#### Genetically engineered Tregs

##### CAR Tregs

**Fig. 4:**
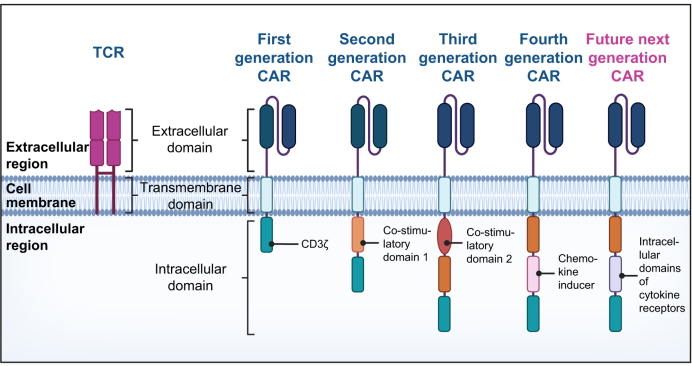
Structural development of several generations of CARs. With each generation, new modifications have been incorporated to enhance the CAR's action. The CAR extracellular antigen binding domains are traditionally based on monoclonal antibody binding sites and are formed of single-chain variable fragments. CAR, chimeric antigen receptor. All figures Created in BioRender. Fleming, M. (2025) https://BioRender.com/q45x981.

The basis of CAR T cell therapy originated during the 1980s as researchers sought to target cancer using modified immune cells.^73^ In CAR T cell therapy, T cells are reprogrammed to express a synthetic receptor that allows them to recognise a predefined antigen in the target cancer cell, independently of MHC molecules. These synthetic receptors are comprised of an extracellular antigen recognition domain (usually a single-chain antibody fragment), and one or more intracellular activation domains. Several generations of CAR constructs have been developed as shown in Fig. 4. First-generation CARs contained a single CD3ζ signalling domain (derived from a TCR); second-generation CARs contain a costimulatory domain (*e.g.* CD28 or 4-1BB) in addition to CD3ζ, while third-generation CARs combine CD3ζ with several costimulatory domains simultaneously. Further engineering steps can result in the addition of functional domains enhancing cell function (intrinsic functions) or equipping the cells with the ability to release factors to alter their environment (extrinsic functions).^74^ The same CAR technology can be employed to generate CAR Tregs. CAR Tregs have generated much enthusiasm not only because of their ability to steer the immune response back towards homeostasis but also because of their potential to supersede the efficacy and capabilities of their un-engineered counterparts. CAR Tregs resolve several limitations observed in other Treg therapies such as antigen specificity, low numbers of precursor cells and lineage stability. CAR Tregs recognising HLA-A2 have entered clinical trials to prevent rejection in HLA-A2-negative transplant recipients who have received an HLA-A2-positive liver or kidney allograft (Table 1).

CAR Tregs are usually generated by transducing Tregs with viral vectors encoding for the CAR construct. Commonly used viral vectors include lentivirus, gamma-retroviral and adeno-associated viral vectors. Alternative methods of engineering cells to express CAR constructs include viral-free systems such as sleeping beauty^75^ or piggyBac transposon which can be used to integrate CAR-encoding DNA into the Treg genome.^6^ CRISPR Cas9 gene-editing technology is another viable method and allows for DNA insertion at specific locations in contrast to viral vectors. Integration of vector DNA in close proximity to an oncogene in the host cell is a potential risk of this strategy, particularly when using viral vectors, although the safety profile of lentiviral vectors is very good when used to transduce T lymphocytes.^76^^,^^77^

At present there are several factors that can be implemented to engineer the next generation of CAR Treg technologies. This includes gaining a more in-depth knowledge of the environment these cells will be subjected to. Extrinsic functions or functional CAR domains were briefly mentioned earlier as a means for CAR Tregs to modulate their surroundings by producing cytokines such as IL-10. Mohensi *et al.* generated HLA-A2-CAR Tregs that co-expressed IL-10. The suppressive capacity of the A2-CAR Tregs was significantly enhanced by the production of IL-10 when co-cultured with A2^+^ B-lymphoblastic cell lines.^78^ This work demonstrated that CAR Tregs could be engineered to co-express IL-10 without impacting Treg phenotype and expansion. Progress has also been made in programming CAR T cells to release specific factors upon sensing molecules associated with tissues and cells in different disease states.^79^

To further develop these therapies in a liver specific context, a greater understanding of the intrahepatic microenvironment in both steady and disease states is needed. Incorporating liver-specific adaptations to CAR Treg therapies can equip these cells with the mechanisms to persist in the liver and execute their immunosuppressive functions with greater efficacy. This is another factor to consider during optimisation of treatment design, as implementation of functional domains previously mentioned may be used to enhance Treg survival in hostile environments.

##### Transduced TCR (tTCR) Tregs

As an alternative to CARs, Tregs can also be engineered to express a synthetic antigen-specific TCR. While this requires the target antigen to be presented by a specific HLA class-II molecule (and therefore limits its use to those patients with specific HLA-II types), the synthetic TCR provides a more physiological type of activation than a CAR. For instance, T cells can be activated via their TCR through as few as one antigen-HLA complex, while CARs require much more antigen binding to facilitate T-cell activation.^80^^,^^81^ This feature may become important when selecting the target antigen. A higher threshold of antigen presence may prevent unwanted or excessive activation, while a lower threshold for activation may be best suited to rarer cell/tissue-specific proteins. In addition to this, a tTCR allows Tregs to act at the secondary lymphoid tissue level, where most antigen presentation takes place. The potential clinical use of tTCR Tregs is being explored in various disease settings including type 1 diabetes and haemophilia A.[82], [83], [84], [85]

##### Ectopic FOXP3-expressing T cells

CD4^+^ Teffs can be transduced with *FOXP3* through a lentiviral vector in order to generate converted Tregs that share many of the properties of *bona fide* Tregs. Due to the high levels of FOXP3, effector cytokine production is suppressed, and the cells exhibit an immunosuppressive profile, both *in vitro* and *in vivo*.^86^^,^^87^ The details surrounding the epigenetic and functional differences between true *bona fide* Tregs and these converted Tregs remain unclear. Likewise, the long-term stability and true suppressive effectiveness of ectopic FOXP3-expressing converted Tregs is not well understood.[88], [89], [90], [91]

As mentioned earlier in this review, *in vitro*-induced Tregs (iTregs) can be generated from conventional T cells. This is achieved under culture with antigen stimulation alongside IL-2 and TGF-β. While phenotypically these iTregs are similar to pTregs and tTregs, iTregs lack the functional stability of their naturally occurring counterparts. This is believed to be the result of incomplete epigenetic changes at Treg-specific genes (*e.g*. demethylation at certain genomic regions of *FoxP3, Cd25* and *Ctla4*). Mikami, Kawakami *et al.* demonstrated that alterations in CD28 stimulation and cytokine levels (IL-2, TGF-β) induced Treg-specific hypomethylation in iTregs.^92^ Such manipulations may improve the stability of converted T cells. Other methods have been explored to induce FOXP3 expression in conventional T cells *in vitro/in vivo,* including the chemical inhibition or knockdown/knockout of FOXP3-repressing cyclin-dependent kinase 8 and 19.^93^

#### Applicability of Treg therapies: Challenges to consider

Advances in cell engineering have facilitated the development of several Treg-based cell therapies capable of migrating to areas of inflammation and acting on a multitude of immune cells associated with inflammation. While these therapies hold a lot of potential, there are still obstacles to their successful application.

There is increasing interest in the efficacy of adoptive cell therapies in autoimmune liver diseases, particularly antigen-specific approaches. That said, the efficacy of antigen-specific therapies hinges on the assumption that there is a suitable antigen to target. The lack of concrete knowledge surrounding disease aetiology results in a less targeted approach. That said, results from the AUTUMN trial, where autologous Tregs labelled with Indium III were administered to patients with AIH, showed that 22-44% of Tregs trafficked to the liver in patients with AIH type I. This study further supports the safety of polyclonal Treg therapies and the likelihood of such Tregs homing predominantly to areas of inflammation. However, the limitations of polyclonal Treg therapies outlined above still apply. Therefore, a tissue targeted rather than a disease-specific approach, through CAR Tregs or tTCR Tregs, may be most suitable.

Furthermore, in the context of liver transplantation, the ARTEMIS trial reported that Treg donor reactivity is decreased following liver transplantation.^100^ This limits the manufacturing efficacy of donor-reactive Tregs. The underlying cause of Treg depletion is unknown.

Clinical trials have shown that the safety profile of non-engineered Tregs is strong. The expectation is that this will apply to CAR Tregs as well, although this still needs to be confirmed. Despite this, the severe side effects associated with CAR T cells are highly unlikely to occur when using engineered Tregs due to their intrinsic anti-inflammatory properties. The manufacture of CAR Tregs is even more complex than that of CAR T cells, due to the need to accurately sort a rare lymphocyte subpopulation from blood, the requirement for prolonged culture periods, and inconsistent product quality depending on the characteristics of the cell donor. What this means is that, even if proven to be clinically efficacious, the high cost of manufacturing autologous Treg therapies at scale might be prohibitive (CAR Treg therapy can cost between $370,000 and $530,000 per patient, excluding hospital costs and additional drugs, in the United States^105^).

These challenges have fuelled interest in producing ‘off the shelf’ or allogeneic CAR Tregs, particularly using induced pluripotent stem cells as starting material. Not only would this approach reduce the cost of manufacturing bespoke products, but it would also limit donor to donor Treg performance variation and delays in receiving treatment. A supply of ‘off the shelf’ CAR Tregs would also increase accessibility to CAR Treg therapy.^106^ Obstacles remain for ‘off the shelf’ cellular therapies, such as the risk of rejection of the CAR Tregs by the host. There are certain modifications that can be made to the engineered Tregs to shield them from immune-mediated destruction. Hu *et al.* genetically altered CAR T cells to no longer express HLA class II to reduce recognition by CD4^+^ T cells that were resistant to CAR T-cell killing.^107^ Additionally, an NK cell inhibitory receptor was incorporated into the CAR T cell to limit NK cell activation.

## Concluding remarks

Unperturbed inflammation in the liver caused by autoimmune disease is associated with an increased risk of tumour development, liver fibrosis and cirrhosis. Likewise, following liver transplantation, excessive immune-mediated inflammation can result in organ damage and thus diminished graft function.

Immunosuppressive strategies are vital to treating patients with autoimmune liver diseases and in preventing graft rejection in transplant recipients. However, current immunomodulatory drugs, when administered long term, can be detrimental to patient health. Long-term immunosuppression also increases the risk of recurring infections and cancer. Furthermore, failure to respond to first- and second-line drugs and chronic rejection damages the graft, which increases the risk of requiring re-transplantation.

Treg-based approaches hold immense potential for the treatment of inflammation within the liver. CAR Tregs have been brought to the frontline of these therapies as they offer the option of antigen specificity, phenotype stability and the incorporation of tissue repair/anti-fibrotic factors.^108^ While some challenges and questions remain, there is still much to explore in the untapped potential of Treg-based therapies.

## Abbreviations

AIH, autoimmune hepatitis: CAR, chimeric antigen receptor; FOXP3, Forkhead-box protein 3; HLA, human leukocyte antigen; iTregs, induced Tregs; NK, natural killer; PBC, primary biliary cholangitis; PSC, primary sclerosing cholangitis; pTregs, peripheral Tregs; TCR, T-cell receptor; Th, T helper; Tregs, regulatory T cells; tTregs, thymic Tregs.

## Financial support

Niloufar Safinia is funded by an MRC Clinician Scientist Award [Grant Ref: MR/Z506588/1].

## Authors’ contributions

Writing - original draft: all authors. Writing - review & editing: all authors. Figure Design: MF with BioRender.

## Conflict of interest

The authors declare that the research in this manuscript was conducted in the absence of any commercial or financial relationships that could be construed as a potential conflict of interest.

Please refer to the accompanying ICMJE disclosure forms for further details.
